# Recycling silver nanoparticle debris from laser ablation of silver nanowire in liquid media toward minimum material waste

**DOI:** 10.1038/s41598-021-81692-9

**Published:** 2021-01-26

**Authors:** June Sik Hwang, Jong-Eun Park, Gun Woo Kim, Hyeono Nam, Sangseok Yu, Jessie S. Jeon, Sanha Kim, Huseung Lee, Minyang Yang

**Affiliations:** 1 Department of Mechanical & Materials Engineering Education, Chungnam National University (CNU), 99 Daehak-ro, Yuseong-gu, Daejeon, 34134 Republic of Korea; 2 Department of Mechanical Engineering, Korea Advanced Institute of Science and Technology (KAIST), 291 Daehak-ro, Yuseong-gu, Daejeon, 34141 Republic of Korea; 3 Visual Display Business, Samsung Electronics, 129 Samsung-ro, Yeongtong-gu, Suwon-si, Gyeonggi-do 16677 Republic of Korea; 4 Department of Mechanical Engineering, Chungnam National University (CNU), 99 Daehak-ro, Yuseong-gu, Daejeon, 34134 Republic of Korea; 5 Department of Mechanical Engineering, The State University of New York, Korea (SUNY Korea), 119 Songdo Moonhwa-ro, Yeonsu-gu, Incheon, 21985 Republic of Korea

**Keywords:** Surface patterning, Mechanical engineering, Design, synthesis and processing, Nanowires

## Abstract

As silver nanowires (Ag NWs) are usually manufactured by chemical synthesis, a patterning process is needed to use them as functional devices. Pulsed laser ablation is a promising Ag NW patterning process because it is a simple and inexpensive procedure. However, this process has a disadvantage in that target materials are wasted owing to the subtractive nature of the process involving the removal of unnecessary materials, and large quantities of raw materials are required. In this study, we report a minimum-waste laser patterning process utilizing silver nanoparticle (Ag NP) debris obtained through laser ablation of Ag NWs in liquid media. Since the generated Ag NPs can be used for several applications, wastage of Ag NWs, which is inevitable in conventional laser patterning processes, is dramatically reduced. In addition, electrophoretic deposition of the recycled Ag NPs onto non-ablated Ag NWs allows easy fabrication of junction-enhanced Ag NWs from the deposited Ag NPs. The unique advantage of this method lies in using recycled Ag NPs as building materials, eliminating the additional cost of junction welding Ag NWs. These fabricated Ag NW substrates could be utilized as transparent heaters and stretchable TCEs, thereby validating the effectiveness of the proposed process.

## Introduction

Silver nanowire (Ag NW) percolation networks have attracted considerable attention as flexible and stretchable transparent conductive electrode (TCE) materials because they have high electrical conductivity and higher flexibility than indium tin oxide (ITO)^1–3^. These advantages have led to the development of various Ag NW-based optoelectronic devices, such as touch screen sensors^4,5^, transparent heaters^6,7^, skin sensors^8–10^, organic light-emitting diodes (OLEDs)^11,12^, and solar cells^13,14^. As the Ag NW layer is usually fabricated from a solution process, patterning of the deposited Ag NW layer is performed for using this layer in practical applications. For example, Shin et al. reported a capacitive touch screen sensor fabricated from a patterned Ag NW layer^5^ and Hong et al. introduced a patterned Ag NW transparent heater for focused heating in specific regions^7^. Local thermal insulation from patterned Ag NW cloth is also reported by Hus et al.^9^, and Won et al. discussed the improved mechanical properties of electronic skin sensors obtained from a Kirigami-engineered pattern of Ag NWs^10^. For patterning Ag NWs, several methods have been introduced, including photolithography^15,16^, printing^17,18^, laser ablation^19,20^, and a transfer method^21,22^. Among the various patterning processes, pulsed laser ablation (PLA) of Ag NWs is considered a promising patterning process because it involves a simple and inexpensive setup without requiring the use of a mask or processes such as chemical etching or lithography. In particular, nanosecond near-infrared PLA (NIR PLA) of Ag NWs has been studied extensively^19,23–28^ because of the relatively low-cost source and little damage to the non-rigid substrate.

One of the major issues of the conventional laser patterning process, which is a subtractive process, is waste generation as patterning is performed by material removal. In the patterning process through laser ablation, the target material irradiated with high energy usually vaporizes or forms a plasma and disappears. As the patterned region grows, the resulting waste also increases, and additional costs are incurred^29^. Furthermore, as the patterned Ag NWs inherently have weak contact bonds, an additional junction welding process is often required to produce high-performance Ag NW TCEs. With the requirement for additional equipment or materials, the advantages of PLA, which are its simplicity and low cost, are easily lost. Therefore, there is a strong need for a laser patterning process that generates less waste and involves simple and low-cost junction welding of the non-ablated Ag NWs.

Pulsed laser ablation in liquid (PLAL) is a simple process that generates nanoparticles from a bulk target material^30–32^. When a focused laser beam is irradiated onto the target material, it experiences plasma formation, followed by fragmentation due to the strong effects of cavitation bubbles. The condensed plumes split, thereby generating NPs. Moreover, surface potentials are formed under the influence of the surrounding liquid media. The resulting NPs can be deposited on conductive electrodes by electrophoretic deposition (EPD), thereby forming metal nanostructures^33–38^.

In this study, we developed a minimum-waste laser patterning process utilizing silver nanoparticle (Ag NP) debris through laser ablation of Ag NWs in liquid media. As the generated Ag NPs can be used for a variety of applications, the fraction of Ag NWs discarded during the typical laser patterning process can be dramatically reduced. Furthermore, from the EPD of the recycled Ag NPs onto unpatterned Ag NWs, enhanced junctions of Ag NWs could be easily formed from the deposited Ag NPs. The unique advantage of this method is that the recycled Ag NPs are used as building materials for fabricating Ag NWs decorated with Ag NPs, so there is nearly no additional cost incurred for junction welding of the Ag NWs. This process is superior in terms of the cost compared to the methods developed in previous research, which used complex processes or additional materials such as light-induced welding^2,39–42^, synthetic methods^43–45^, chemical soldering^46,47^, electrochemical metal deposition^48–51^, and polymer coating^45,52,53^. Through PLAL and EPD, a patterned substrate of Ag NWs decorated with Ag NPs was formed and used as a transparent heater and stretchable TCE, confirming that the process is suitable for practical applications.

## Results and discussion

**Figure 1:**
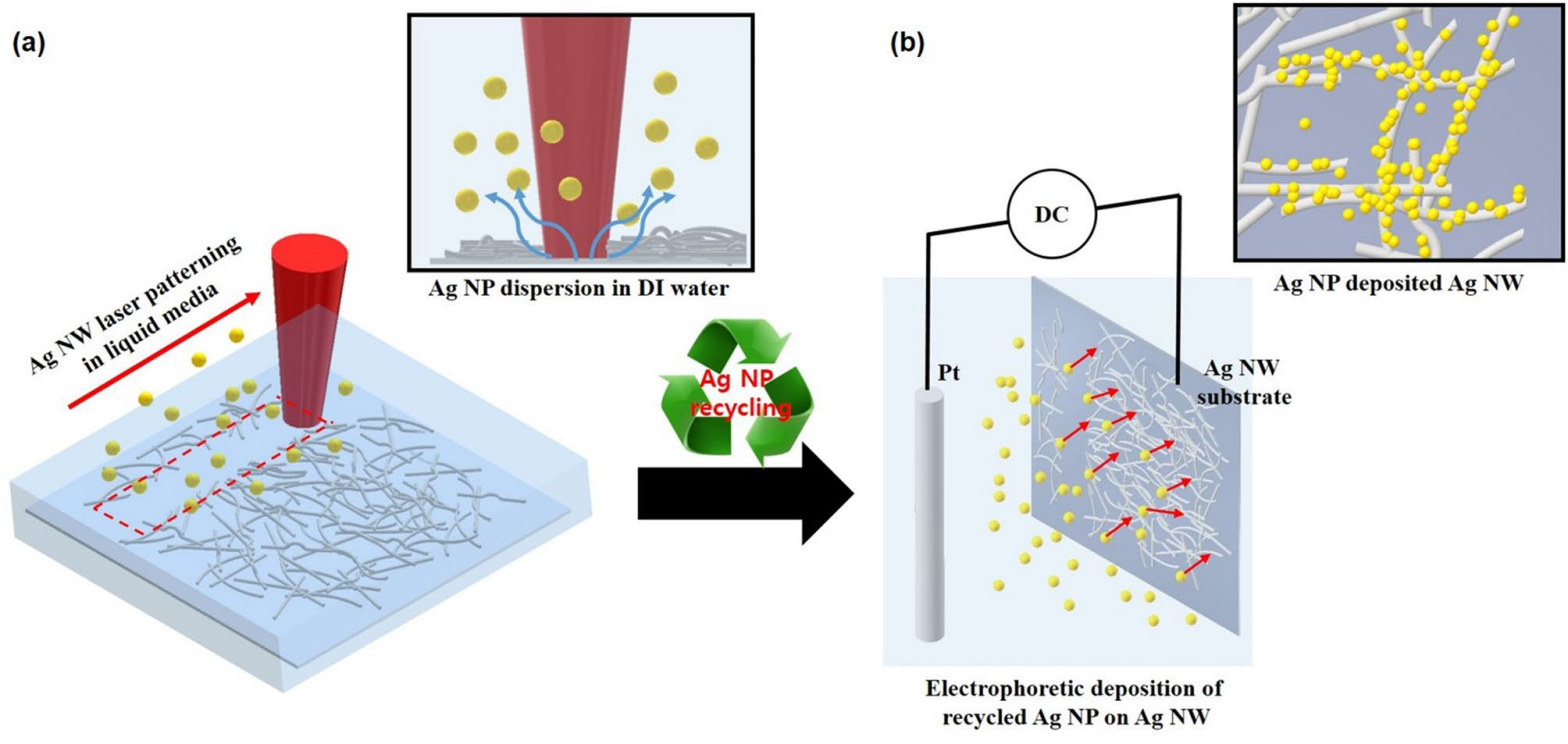
Schematic of the minimum-waste laser patterning process. (**a**) Pulsed laser ablation (PLA) of silver nanowire (Ag NW) in liquid media. The inset presents the dispersion of Ag NP debris. (**b**) Electrophoretic deposition (EPD) of silver nanoparticles (Ag NPs) on non-ablated Ag NWs. Inset shows the junction-enhanced Ag NW from Ag NP deposition.

Figure 1 shows the schematic of the minimum-waste laser patterning process. In the water-assisted laser ablation of Ag NWs, the Ag NP debris, which should be vaporized under ambient conditions, remained in the liquid media. While conventional water-assisted laser ablation has been used to remove surface residues^54–56^ or produce metal nanoparticles^57,58^, laser patterning and production of nanoparticles can be conducted simultaneously in the proposed process (Fig. 1a). The nanoparticles (NPs) can be deposited onto the electrode by electrophoretic motion via application of an external electric field. When the electric field is applied to a platinum electrode and a patterned Ag NW substrate, the Ag NWs act as the working electrode. Accordingly, Ag NPs were deposited on the non-ablated Ag NW layer, as shown in Fig. 1b. Because the deposited Ag NPs enhanced the contact bonding of the Ag NWs, a lower contact resistance and increased robustness could be obtained for Ag NWs decorated with Ag NPs.

**Figure 2:**
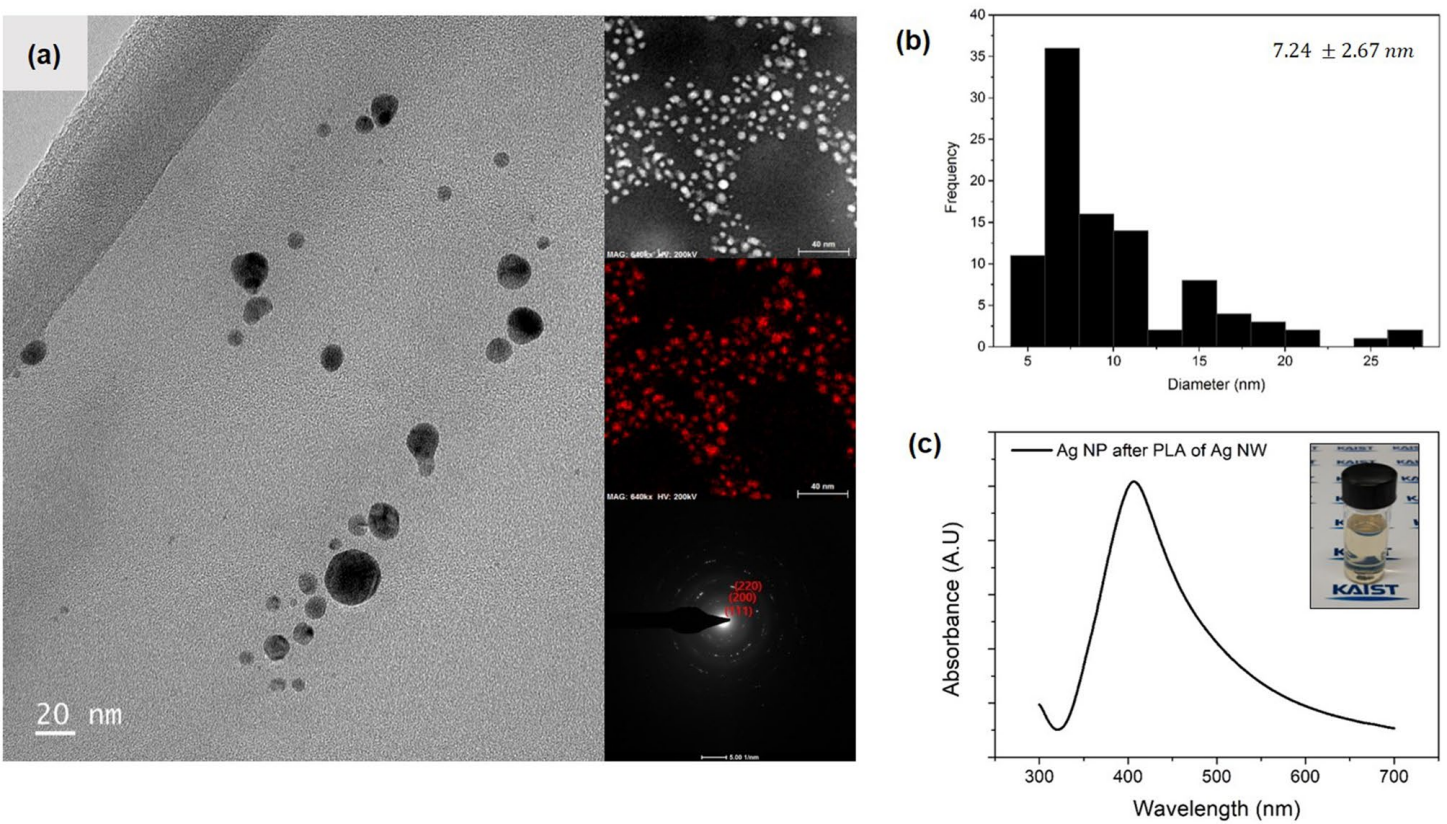
Characteristics of Ag NPs generated from PLA of Ag NW in liquid media. (**a**) Transmission electron microscopy (TEM) image of Ag NPs. The inset presents the elemental maps and selected area electron diffraction (SAED) pattern. (**b**) Size distribution of Ag NPs. The median size and standard deviation were calculated from Gaussian fitting. (**c**) Absorbance of the Ag NP solution. The inset shows the yellowish color of the Ag NP solution.

As the application of metal NPs depends on their properties, the morphology and elemental composition of the generated Ag NPs were analyzed. Figure 2a shows a transmission electron microscopy (TEM) image depicting generally spherical and well-dispersed Ag NPs. Energy dispersive spectrometer (EDS) measurements showed that the main component of the NPs was silver. It is known that a thin oxide film of several nanometers is usually formed from the LAL of a silver substrate^59^. However, no severe surface oxidation was observed during our measurements (Figs. S1 and S2). Silver was detected as the prominent element owing to the relatively long penetration depth of XRD as reported in previous studies^60^. Selected area electron diffraction (SAED) measurements also revealed that the crystallinity was consistent with the face-centered cubic structure of silver^61^. To identify the size distribution, the TEM images of 100 samples were selected. The diameters of the NPs were calculated through image processing as shown in Fig. 2b. Although some of the NPs had sizes larger than 20 nm because of the physical synthesis involved, most of the particles were found to have a size less than 10 nm. From Gaussian fitting, the mean and the standard deviation of the sizes were observed to be 7.24 nm and 2.67 nm, respectively. The absorbance and color properties of the NP solution were typical of Ag NPs. From Fig. 2c, it can be observed that the solution has an absorbance peak around 400 nm, which is identical to the plasmon resonance absorption peak of Ag NPs^62^. A yellowish color, a specific characteristic of Ag NP colloids, was also observed as shown in the inset. The main difference between the Ag NW layer and a conventional bulk silver plate during the LAL is that the Ag NPs produced from the former exhibited superior properties compared to the bulk target in terms of morphology and oxidation. While Ag NPs formed from the Ag NW layer maintained a dispersion of 10 nm or less, the NPs were often aggregated in the bulk target (Fig. S3a). Figure S3b shows that even the chain shapes of the connected NPs were because of intensified aggregation under the same processing conditions used for Ag NW LAL. It was assumed that the different processing results were due to the amount of the ablated material and the shape of the cavitation bubble. In the case of the bulk target, aggregation can be easily caused because increased material interaction often occurs within the cavitation bubble formed by the relatively expanded ablated target material^30^. Moreover, when a metal wire is ablated in a liquid, the unconventional shape of the cavitation bubble induces quick detachment of the bubble from the target^63,64^. Therefore, an improved morphology of Ag NPs was generated from reduced material interaction and the shape of the cavitation bubble. The Ag NPs formed from Ag NWs were less affected by surface oxidation. Figure S3e shows the EDS results of the Ag NPs produced from the Ag plate, indicating that oxygen was detected on the surface of the NPs, which is easily distinguishable from the surrounding oxygen. When comparing the oxygen content along the line profile (Fig. S3f), it was found that the Ag NPs from Ag NWs were less affected by oxidation.

**Figure 3:**
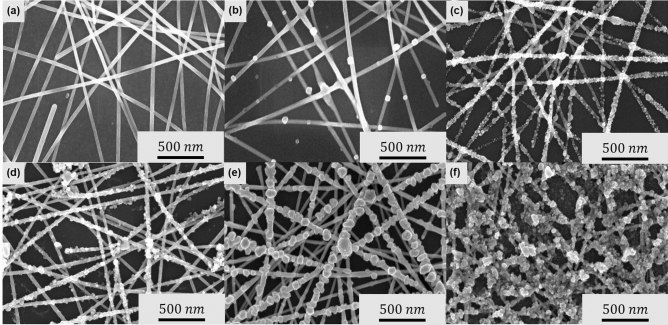
Morphological change of Ag NWs after EPD of recycled Ag NPs. Scanning electron microscopy (SEM) images of (**a**) pristine Ag NW and EPD-processed Ag NWs at (**b**) 1 V/cm, (**c**) 2.5 V/cm, (**d**) 5 V/cm, (**e**) 7.5 V/cm, and (**f**) 10 V/cm.

To fabricate the Ag NWs decorated with the generated Ag NPs, the EPD process was utilized. The electrophoretic motion of Ag NPs according to the applied external voltage can be expressed through Henry's equation as follows^65^:

1 $$\upmu = \frac{\nu }{E}= \frac{2}{3} \frac{{\varepsilon }_{0}{\varepsilon }_{r}\xi }{\eta }\mathrm{ f}\left(\mathrm{\kappa r}\right),$$

where ε_0_ is the vacuum permittivity, ε_r_ and *η* are the relative permittivity and viscosity of the liquid, respectively, and f(κr) is a function depending on the radius (r) and the double layer thickness (κ^−1^). The movement of NPs within the electric field is mainly affected by the electric field and the zeta potential of the NPs. As the generated NPs have a positive zeta potential (Fig. S4), a cathodic EPD process was performed. Figure 3 shows the surface morphologies of pristine Ag NWs and after performing the EPD process for 10 min. The pristine Ag NW layer had a diameter of approximately 20 nm and exhibited a typical nanowire percolation network with weak contact bonding. However, after the EPD of Ag NPs at 1 V/cm, partial deposition of Ag NPs with a diameter of approximately 40 nm was observed. In particular, owing to the aggregation of Ag NPs on the contact area, it was found that an enhanced junction was effectively formed even under relatively low voltage conditions. At 2.5 V/cm, the EPD of the NPs was actively conducted to form Ag NWs decorated with Ag NPs. As the voltage increased, the Ag NPs gradually deposited on the entire surface area of the NWs. Larger Ag NPs were formed from the aggregated Ag NPs. At 5 V/cm, the morphology was similar to that obtained at 2.5 V/cm. However, aggregation was observed in some regions decorated with Ag NPs having a diameter above 100 nm. Above an electric field of 7.5 V/cm, large lumps of Ag NPs (100–200 nm diameter) were formed as many Ag NPs were deposited simultaneously. Finally, at 10 V/cm, the surface of the Ag NWs was completely covered by the Ag NPs. The diameter of the Ag NPs exceeded several hundreds of nanometers in this condition. Owing to the enhanced junction of Ag NWs, the Ag NWs decorated with Ag NPs have better mechanical and electrical properties than pristine Ag NWs. However, it is essential to select the appropriate voltage conditions because the transparency decreases as the deposited Ag NPs occupy a larger area.

**Figure 4:**
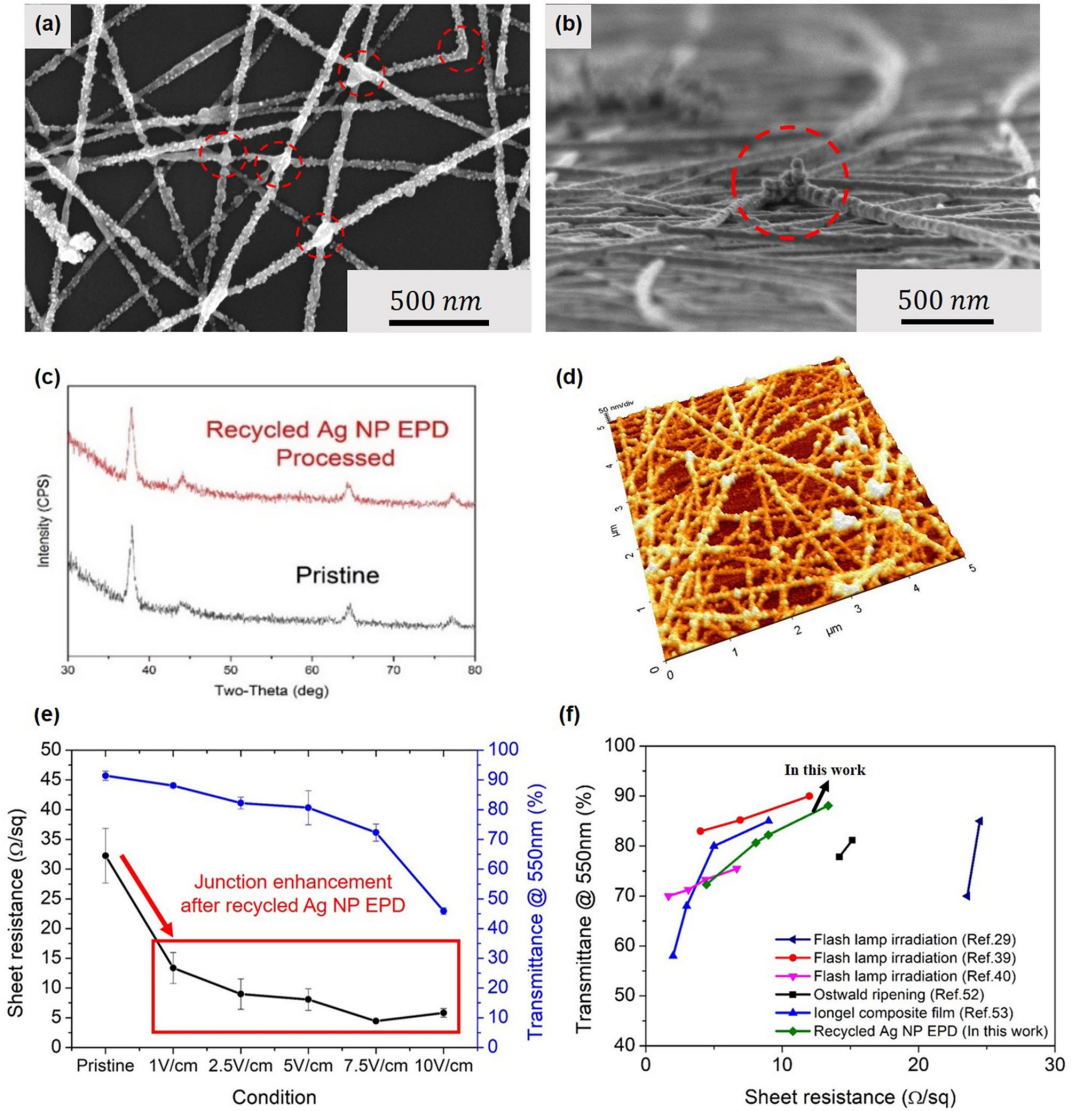
Surface properties and performance of junction-enhanced Ag NWs. (**a**) Plane image and (**b**) tilted SEM image of EPD-processed Ag NWs. (**c**) XRD spectrum before and after the EPD process. (**d**) Atomic force microscopy (AFM) image of Fig. (a). (**e**) Sheet resistance and transmittance of Ag NWs at 550 nm at different electric fields. (**f**) Performance comparison between EPD-processed Ag NWs from recycled Ag NPs and previously reported junction-welded Ag NWs.

As the performance of Ag NWs usually depends on the morphology and surface properties, the characteristics of the EPD-processed Ag NWs were analyzed. Figure 4a shows a scanning electron microscopy (SEM) image of the Ag NWs after EPD at 2.5 V/cm. It was found that Ag NPs were deposited along the Ag NWs, forming Ag NWs decorated with Ag NPs. In particular, as shown in the red dashed area, aggregated Ag NPs were formed in the contact spots of Ag NWs. An enhanced junction due to the deposition of Ag NPs was expected to lower the contact resistance and increase the mechanical reliability by increasing the contact bonding of Ag NWs. The tilted SEM image in Fig. 4b shows that strongly bonded Ag NWs are formed from the aggregation of Ag NPs at the junction region. From the component analysis of the particles attached to the Ag NWs (Fig. S5), it was confirmed that the recycled Ag NPs were the key materials that strengthened the junction of the Ag NWs. As shown in Fig. 4c, XRD analysis before and after the EPD process also identified that the generated Ag NPs were the causes of junction enhancement. As no silver oxide peaks were detected, it was concluded that degradation due to surface oxidation of Ag NPs was negligible. This result was similar to those of various other studies on junction welding of Ag NWs processed in liquid media^48,66^. The surface properties of the Ag NWs decorated with Ag NPs were revealed by atomic force microscopy (AFM) measurements, as shown in Fig. 4d. After the EPD process, it was found that the Ag NWs had a rough surface on which particles were agglomerated and bonded to each other. As the electric field was increased, the surface roughness of the Ag NWs was also increased from the additional deposition of Ag NPs. As shown in Fig. S6, the pristine Ag NWs are tightly clustered with relatively weak contact bonding. With the application of the electric field (2.5 V/cm), the particles attached along the NWs, thereby forming Ag NWs decorated with Ag NPs. As the electric field was increased up to 5 V/cm, the aggregation of Ag NPs intensified, forming a strong bond between the Ag NWs. However, an excessive electric field induces rapid growth of NPs, which often deteriorates the transparency of the Ag NW substrate. The changes in the sheet resistance and transmittance were measured at different values of the applied electric field. Figure 4e shows the sheet resistance and the transmittance of Ag NWs at 550 nm under different electric fields. When EPD of the Ag NPs was performed, a noticeably decreased contact resistance was observed. At 1 V/cm, the sheet resistance of the Ag NWs decreased from 32.26 Ω/sq to 13.36 Ω/sq. As the electric field was increased from 1 to 10 V/cm, the sheet resistance decreased to below 10 Ω/sq. It was believed that this improvement was due to the increased deposition of Ag NPs on the Ag NWs. However, as the deposition of Ag NPs increased, the transparency decreased owing to the increased scattering by Ag NPs (Fig. S7). Pristine Ag NW/glass, after vacuum filtration, normally has a transmittance of 91% at 550 nm. As the applied electric field increased from 1 to 10 V/cm, the transmittance value reduced from 89.05 to 47.52%. Therefore, in the proposed process, 1 V/cm with excellent transmittance (89.05% at 550 nm) and low sheet resistance (13.36 Ω/sq) was selected as the optimal condition to produce Ag NW TCEs. Figure 4f shows the performance comparison of the Ag NP EPD process with processes used in previous junction welding studies. Although it was not superior, a comparable level of performance improvement was achieved. The unique advantage of the Ag NP EPD process is that little additional cost is needed to produce the enhanced junction of Ag NWs. When patterning junction-welded Ag NWs, welding and patterning are often performed separately^39,67^. On the other hand, as the recycling process utilizes the by-products generated from the laser patterning process, patterning and junction strengthening can be performed in a simple and inexpensive way. Therefore, the proposed process is superior to previous methods in terms of efficiency and eco-friendliness.

**Figure 5:**
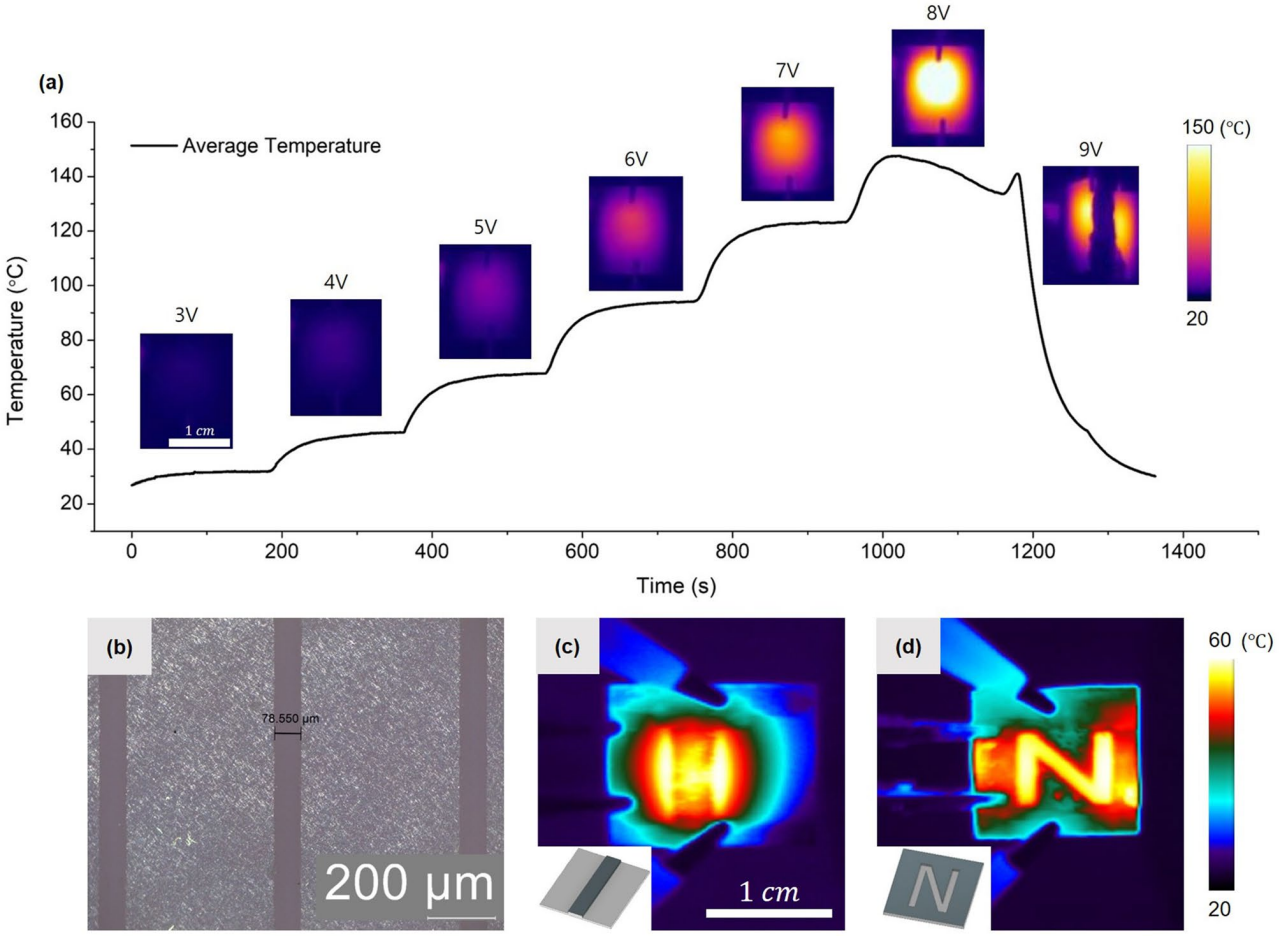
Ag NW transparent heater demonstration. (**a**) Transient temperature evolution of the Ag NW heater at stepwise voltage increases from 3 to 9 V. (**b**) Optical image of patterned Ag NWs after PLA. Selective heating of specific region from patterned Ag NWs; IR image of Ag NWs with the (**c**) embossed line and (**d**) engraved “N” shape. The insets present the ablation regions.

The junction-enhanced Ag NW layer can be utilized in various optoelectronic devices. To demonstrate the usability of the proposed process, a substrate of Ag NWs decorated with Ag NPs was fabricated as a transparent heater and its performance was tested. The test was conducted using the method for evaluating the performance of Ag NWs as transparent heaters in previous studies^1,6,7^. Figure 5a shows an IR camera image when a DC voltage is applied to the pristine Ag NW/glass substrate after the EPD at 1 V/cm for 10 min. When the voltage increased from 3 to 9 V at 3-min intervals, the temperature gradually increased and converged to a constant temperature under the given conditions. At 8 V, the temperature increased up to 150 $^\circ \mathrm{C}$ Although the temperature decreased after the peak temperature, the performance was not attributed to deterioration from the degradation of the Ag NWs due to surface oxidation of Ag NWs, which has been reported to occur at higher temperatures^1^. Instead, the degradation appeared to be the effect of substrate damage due to thermal stress from the rapid temperature rise. Under the 9 V condition, the substrate broke into two pieces as the thermal stress was intensified. The high temperature profile shows that Ag NWs decorated with Ag NPs can be effectively utilized as transparent heaters. For practical applications of Ag NW TCEs, the patterning process is important depending on the end use. When patterned Ag NWs are used as transparent heaters, the temperature can be controlled selectively as the current flow is concentrated in the non-ablated region^7,68^. The patterned Ag NWs decorated with Ag NPs prepared from the proposed process were evaluated to determine their capability for offering selective temperature control. During laser patterning of Ag NWs, an arbitrary shape could be produced using a galvanometer scanner with a line width of ~ 78 μm (Fig. 5b). Figure S8 shows the optical image of the patterned Ag NW substrate with (a) an embossed line and (b) an engraved “N” shape. Transparent electrodes with desired shapes can be easily patterned through laser ablation. After the laser ablation process, the EPD of recycled Ag NPs was also conducted. Figure S5c and 5d show the IR images of the patterned substrate. Under the 5 V condition, it was found that the temperature of the substrate rose to ~ 60 $^\circ \mathrm{C}$. In addition, due to a concentrated current density, significantly higher temperatures occurred in the non-ablated Ag NWs compared to the surrounding substrate. Accordingly, through a simple laser patterning process and junction enhancement through EPD, a transparent heater utilizing Ag NWs, which provided selective temperature control, of the desired shape was easily formed.

**Figure 6:**
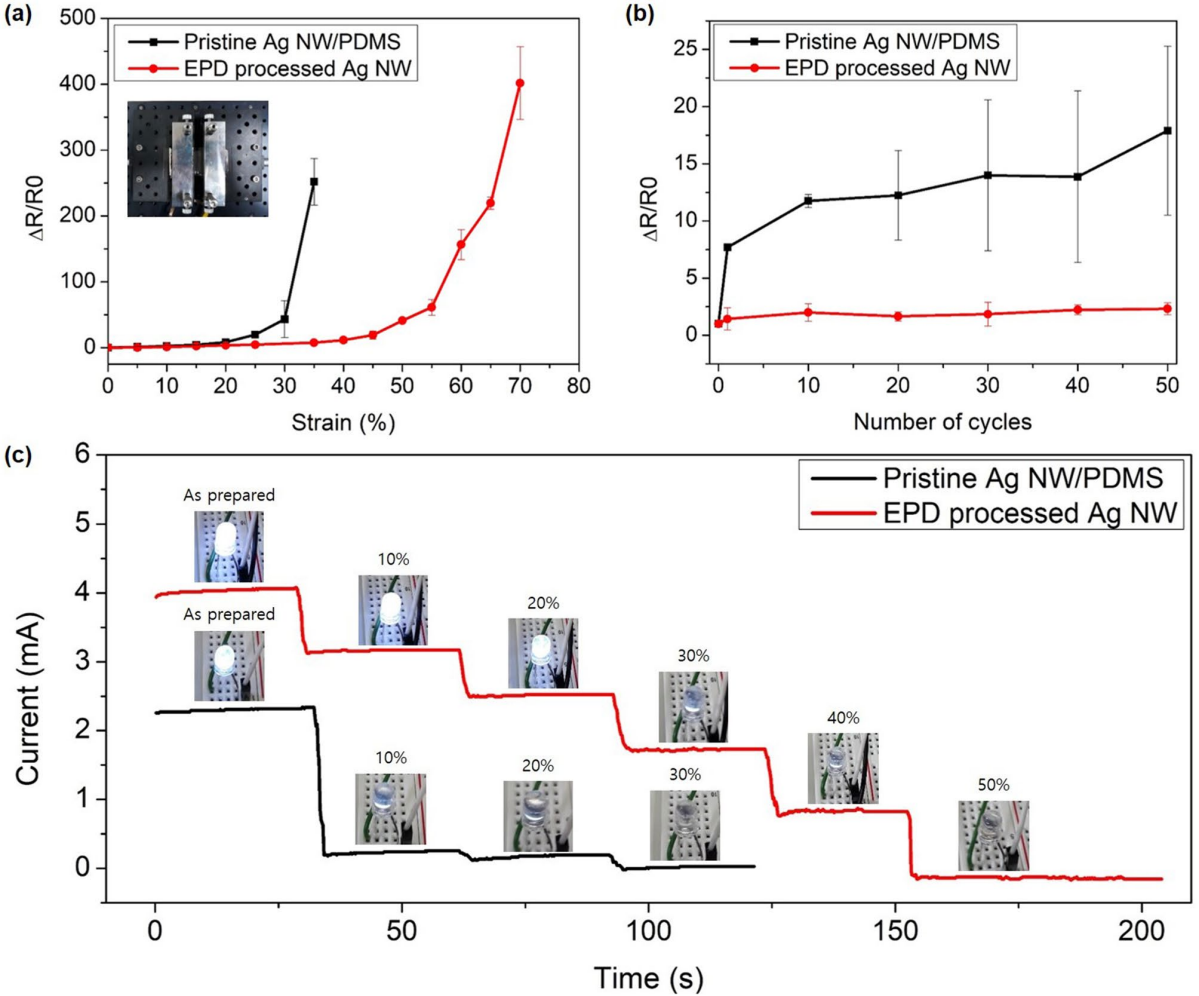
Electromechanical properties of junction-enhanced Ag NW/PDMS. The resistance changes in the pristine and EPD-processed Ag NWs depending on (a) the stretching strain and (b) repetitive cycles at 20% strain. (c) The change in currents in pristine and EPD-processed Ag NWs connected to an LED. Images of the LED at strains varying from 10% to 50% (3 V).

The flexibility of Ag NWs, which enables them to operate as TCEs on non-rigid substrates, is one of the main reasons for their preferred use over ITO. To examine whether the proposed process can be applied to stretchable substrates, Ag NWs were transferred to polydimethylsiloxane (PDMS), following which EPD of the recycled Ag NPs was performed. Decreased resistance and transmittance values were observed showing that the experimental results were similar to those obtained for the glass substrate. After the EPD at 1 V/cm for 10 min, the resistance decreased from 126.8 Ω to 72.9 Ω, and transmittance at 550 nm decreased from 88 to 85% (Fig. S9). Figure 6a shows the change in resistance of pristine Ag NWs and EPD-processed Ag NWs depending on the strain. The strain was adjusted by a micrometer while keeping both ends of the Ag NW/PDMS fixed as shown in the inset. On comparing the changes in resistance values, the EPD-processed Ag NW layer exhibited better electrical performance than pristine Ag NWs. In the case of pristine Ag NWs, the resistance change was relatively less until 20% strain was reached and increased rapidly from 25 to 40% strain, beyond which the resistance was not measured. On the other hand, the resistance change of EPD-processed Ag NWs was not substantial up to 45% strain, and the resistance was measured up to 70% strain even after a considerable increase in resistance had occurred. The resistance change ratio was measured as 14.47 at 40% strain. Similar results were obtained when the test cycle was performed under the 20% strain condition (Fig. 6b). The EPD-processed Ag NWs exhibited a relatively small resistance change ratio (2.68) throughout the test cycle, whereas the resistance of pristine Ag NWs increased continuously due to the relatively weak contact bonding of Ag NWs. With repeated numbers of cycles, the difference in the resistance change ratio widened. Therefore, the fabricated Ag NWs decorated with Ag NPs showed better performance as a stretchable TCE than pristine Ag NWs. Finally, patterned Ag NWs decorated with Ag NPs from the proposed process were examined as a stretchable TCE using the LED test. An Ag NW layer transferred onto PDMS (226.22 mg/m^2^) was irradiated with the same fluence as that used for the glass substrate, i.e., 6.31 J/cm^2^, and no PDMS substrate damage was observed. The EPD of Ag NPs was performed on the patterned Ag NW substrate as shown in Fig. S10. The Ag NW substrate was connected in the middle of the LED circuit, and the LED was turned on by applying a voltage of 3 V. Figure 6c shows a comparison of the LED currents for different values of strain on the Ag NW/PDMS substrate. It was observed that junction-reinforced Ag NWs emitted light under strains up to 40%, but pristine NWs did so up to only a 20% strain. On comparing the electric current and brightness between junction-reinforced and pristine NWs for the same strain (20%), the junction-reinforced Ag NWs were found to exhibit improved performance. In another application, we investigated whether the fabricated substrate could operate as a stretchable heater. As shown in Fig. S11, the temperature increases up to 40 $^\circ \mathrm{C}$ under a voltage of 3 V. Additionally, the substrate could operate even under a strain of 20%. Accordingly, it was confirmed that the Ag NWs decorated with Ag NPs could be used as a superior stretchable TCE.

In summary, we propose a minimum-waste laser patterning process by recycling Ag NP debris resulting from the laser ablation of Ag NWs in liquid media. With the application of liquid media, the Ag residues, which would normally be discarded, remained well dispersed in an Ag NP solution. The generated Ag NPs showed superior performance in terms of morphology and oxidation resistance compared to the Ag plating formed via LAL. Through the EPD of Ag NPs, junction-enhanced Ag NWs decorated with Ag NPs were formed, leading to improved electrical and mechanical performances of non-ablated Ag NWs. EPD-processed Ag NWs can be used as transparent heaters with selective temperature control of the laser-patterned Ag NWs. The Ag NW layer transferred onto PDMS could withstand higher strains and a larger number of test cycles than pristine Ag NWs, showing that the former can be used as superior stretchable TCEs. As the proposed process improves the performance of the Ag NWs by utilizing ablated debris that is normally discarded during the laser patterning process, it is more environmentally friendly and economical than the conventional junction welding process.

## Methods

### Vacuum filtration of Ag NWs on a substrate

A substrate was prepared for the transfer of Ag NWs. A 1-in soda-lime glass substrate (SL.Sli1012, SciLab) was used for the characterization and transparent heater application of Ag NWs decorated with Ag NPs. The PDMS substrate used for testing the stretchable TCE was prepared by mixing the PDMS solution and curing agent (Sylgard 184, Dow Corning) in a 10:1 ratio. The mixture was heated at 60 $^\circ \mathrm{C}$ for 3 h. Ag NWs with a diameter of 21 ± 3 nm and length of 22 ± 5 μm were purchased (Flexiowire 2020C, Flexio). The Ag NW percolation network layer was transferred by vacuum filtration^7,69^. Nylon (7402-004, Whatman) and polytetrafluoroethylene (PTFE) filters (190455-1LSC, STERLITECH) were placed on a microassay holder. While maintaining vacuum, 10 ml of isopropyl alcohol (IPA) (HPLC grade, Sigma-Aldrich) with the Ag NW solution was poured into the PTFE filter. The areal densities of Ag NWs were 113.11, 169.67, and 226.22 mg/m^2^ for glass, PDMS, and patterned PDMS substrates, respectively. The substrate (glass/PDMS) was placed in the filter for 1 min so that the Ag NWs could be in contact with the substrate. Thereafter, the vacuum was released, the substrate was gently removed from the filter, and an Ag NW percolation network on the substrate was formed.

### Laser patterning of Ag NWs

To fabricate patterned Ag NWs, an ytterbium fiber laser with a wavelength of 1060 nm (YLD-D30, Han's Laser Technology Co., Ltd; pulse width = 200 ns, repetition rate = 20 kHz, output power = 24 W) was used. It was focused using an f-theta lens with a focal length of 150 mm and a two-dimensional galvanometer scanner. In the line patterning process, the scan speed was 100 mm/s, and in the area patterning process, such as text patterning, the hatch of the line pattern was set to 0.05 mm so that the entire area was removed. The laser ablation of Ag NWs in the liquid media was prepared by carefully dipping Ag NWs in DI water (LC–MS Grade, Merck) contained in a Petri dish (DU.2175541, Duran). In the immersed condition, the substrate was irradiated with a fluence of 6.31 J/cm^2^. After the patterning process, the patterned Ag NW substrate was gently removed using tweezers, followed by blowing with nitrogen gas. A yellowish Ag NP solution was found in the Petri dish after the irradiation. The generated Ag NPs were transferred to a beaker and used for the EPD.

### EPD of Ag NPs onto the unpatterned Ag NWs substrate

The Ag NPs generated via recycling were subjected to EPD. A common power supply (U8001A, KEYSIGHT) was used to apply a constant electric field, which was controlled by varying the voltage and distance between the substrates. The distance between the substrates was fixed at 1 cm, and the electric field was changed by increasing the voltage from 1 to 10 V. For cathodic EPD, the cathode was connected to the patterned Ag NW substrate, and the platinum electrode (CE-POST-Pt-01-05-PTFE-06-80, WIZMAC) was connected to the anode. After the two electrodes were immersed in the Ag NP solution, EPD was conducted for 10 min through voltage application. After EPD, the Ag NW substrate was rinsed with deionized water (DI water), followed by gentle blowing with nitrogen gas.

### Characterization

TEM (Talos F200X) was used to examine the Ag NP debris after PLA of Ag NWs in liquid media. The particle size was calculated from 100 TEM images using the Image J software. The mean size and the standard deviation were obtained by fitting the size frequency diagram to a Gaussian function (Origin 8.1). The absorbance of the generated Ag NPs and transmittance of the Ag NWs were measured using an ultraviolet–visible spectrometer (Evolution 220, Thermo Fisher Scientific), and the zeta potential was measured using Zetasizer Nano ZS (Malvern Panalytical). The surface morphologies of the Ag NWs were identified from SEM (NOVA 230, FEI) and AFM (XE-100, Park Systems). The sheet resistances were measured using a 4-point probe (FPP-400, DASOLENG). The components of the Ag NPs and Ag NWs were analyzed using an EDS and XRD (X’Pert-PRO MRD, PANalytical). For real-time temperature measurements of the stretchable Ag NW TCE, an infrared IR camera (A35, FLIR) was used.

## Supplementary Information

Supplementary Information
