# Supplementary material for: Recycling silver nanoparticle debris from laser ablation of silver nanowire in liquid media toward minimum material waste
Source: Sci Rep. 2021 Jan 26;11:2262. doi: 10.1038/s41598-021-81692-9 (PMC7838405; doi:10.1038/s41598-021-81692-9)
Supplement: Supplementary file 1 — Supplementary Information [file 41598_2021_81692_MOESM1_ESM.docx]

Supporting Information

Recycling Silver Nanoparticle Debris from Laser Ablation of Silver Nanowire in Liquid Media toward Minimum Material Waste

June Sik Hwang^1^, Jong-Eun Park^2^, Gun Woo Kim^3^, Hyeono Nam^2^, Sangseok Yu^4^, Jessie S. Jeon^2^, Sanha Kim^2^, Huseung Lee^1^*, and Minyang Yang^5^*

^1^Department of Mechanical & Materials Engineering Education, Chungnam National University (CNU), 34134, 99 Daehak-ro, Yuseong-gu, Daejeon, Republic of Korea

^2^Department of Mechanical Engineering, Korea Advanced Institute of Science and Technology (KAIST), 34141, 291 Daehak-Ro, Yuseong-Gu, Daejeon, Republic of Korea

^3^Visual display business, Samsung Electronics, 16677, 129 Samsung-ro, Yeongtong-gu, Suwon-si, Gyeonggi-do, Republic of Korea

^4^Department of Mechanical Engineering, Chungnam National University (CNU), 34134, 99 Daehak-ro, Yuseong-gu, Daejeon, Republic of Korea

^5^Department of Mechanical Engineering, The State University of New York, Korea (SUNY Korea), 21985, 119 Songdo Moonhwa-Ro, Yeonsu-Gu, Incheon, Republic of Korea

*Corresponding author e-mail: liber@cnu.ac.kr, minyang.yang@sunykorea.ac.kr


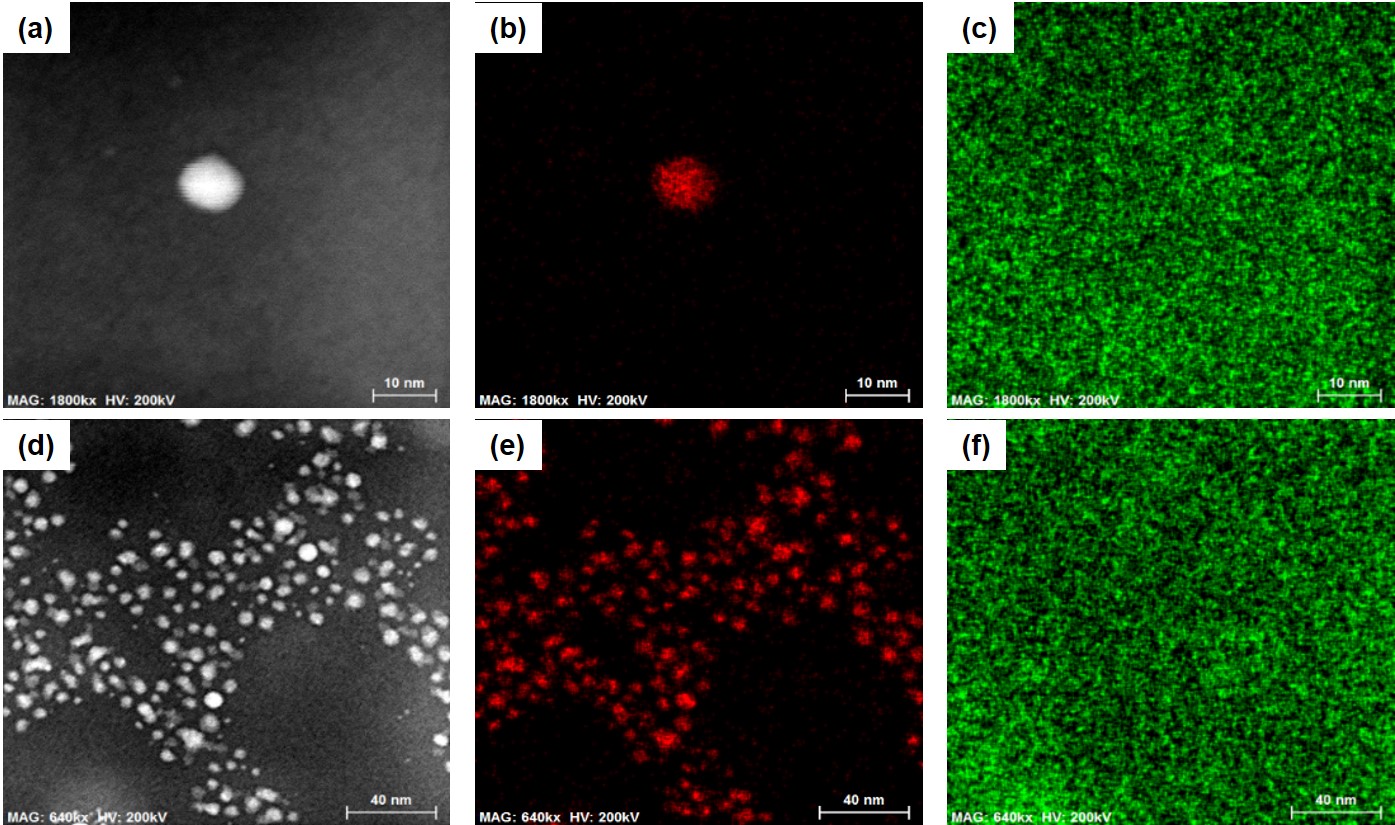


**Figure S1**. STEM elemental maps for generated Ag NPs. (a, d) HAADF image of Ag NPs, (b, e) Ag, (c, f) O.


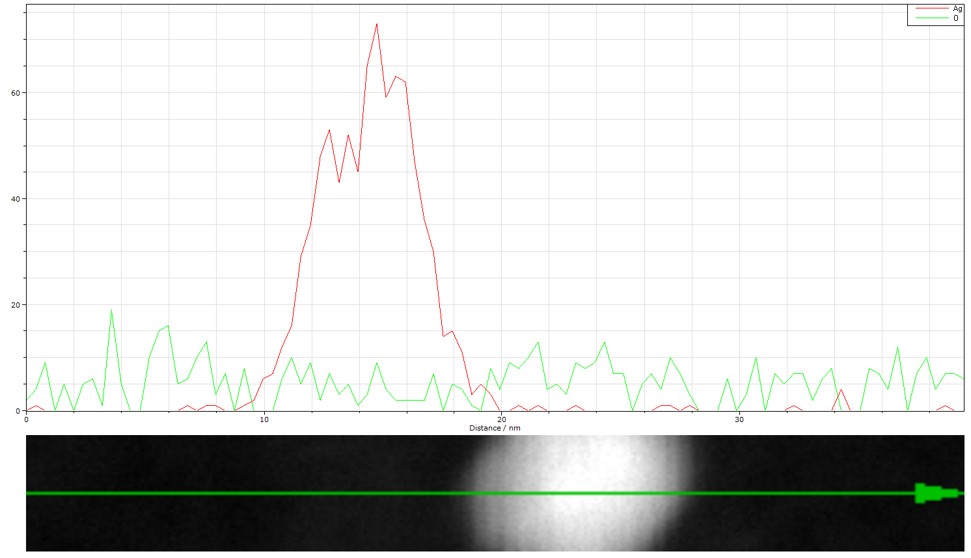


**Figure S2**. Line-scanning profile of Ag NP in the STEM image.


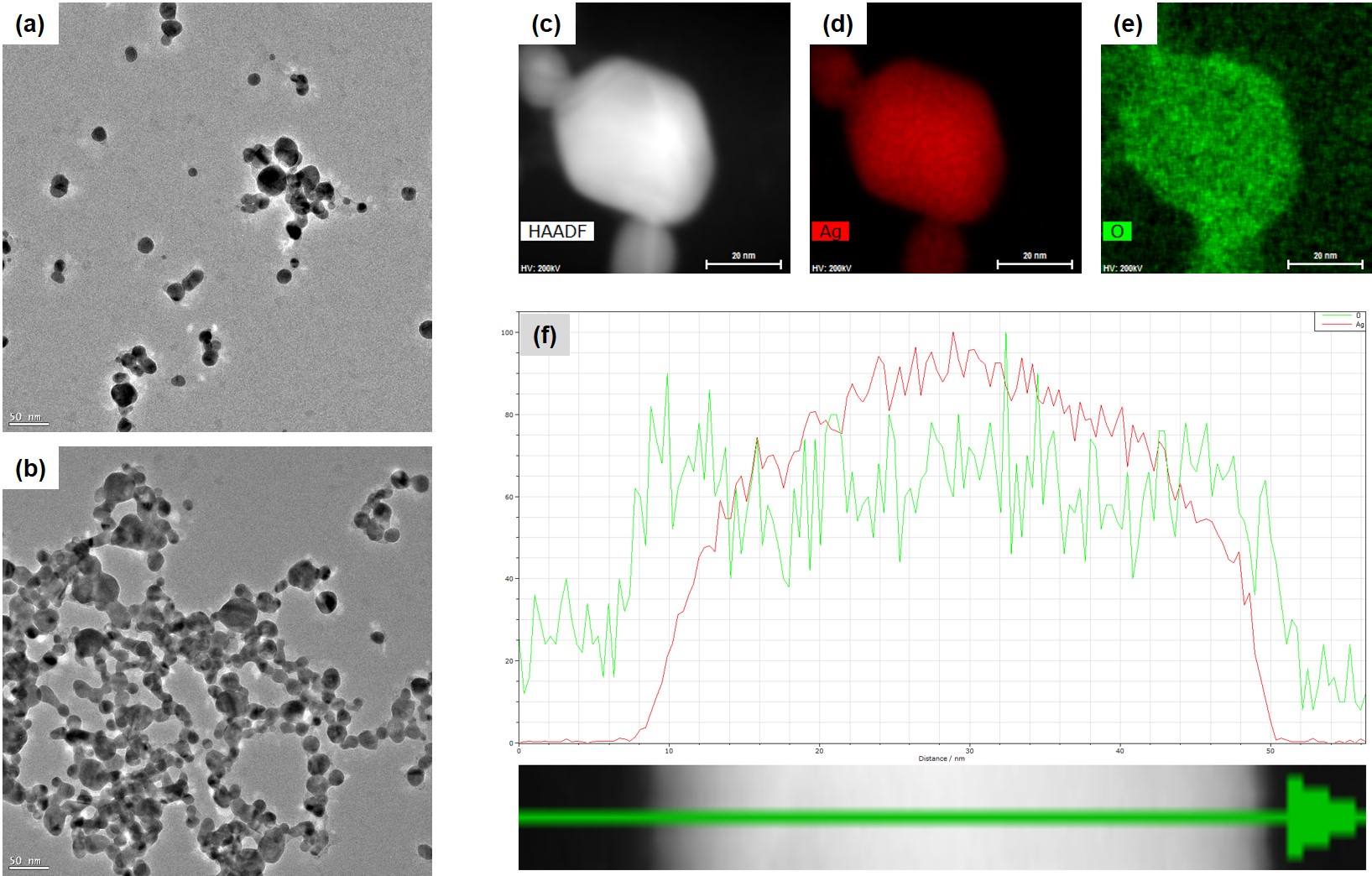


**Figure S3**. Characteristics of generated Ag NP from PLA of bulk Ag plate in liquid media. TEM images of (a) the aggregated Ag NPs, (b) the elongated Ag NP chain. (c) HAADF image, (d) Ag, (e) O image of STEM elemental maps. (f) . Line-scanning profile of Ag NP in the STEM image.


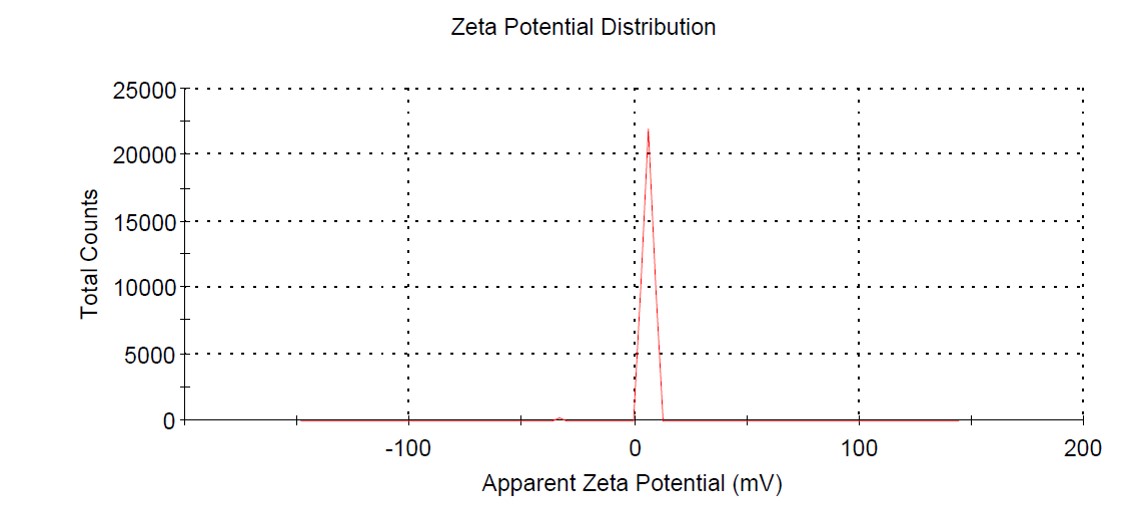


**Figure S4**. Zetapotential of generated Ag NPs.


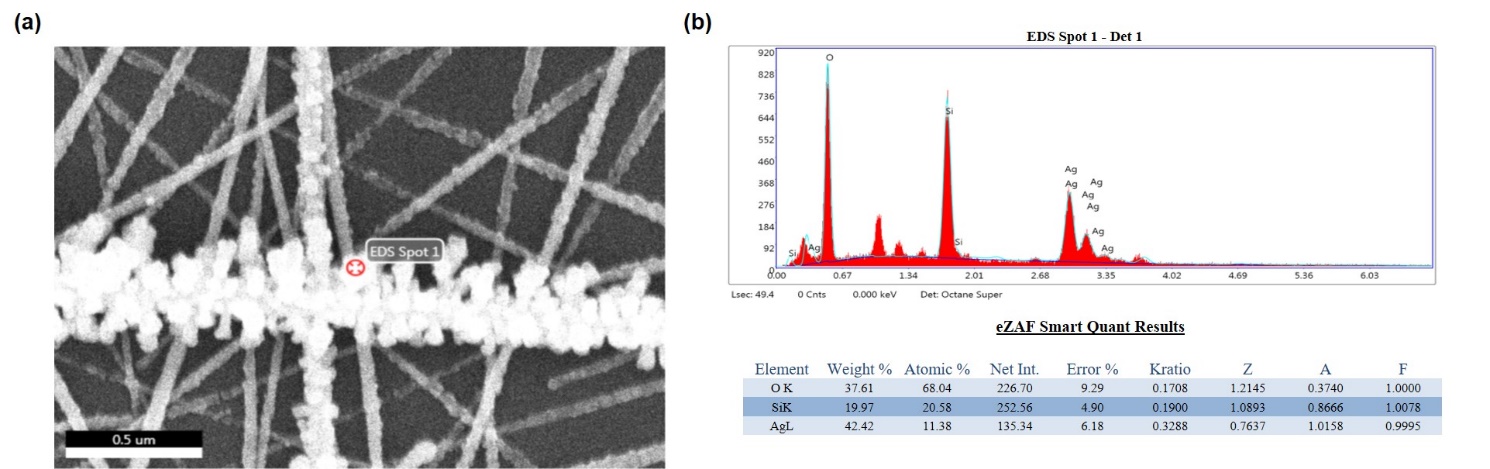


**Figure S5**. EDS of deposited Ag NPs on Ag NW. (a) EDS measurement and (b) elemental spectrum and ratio of deposited Ag NPs on Ag NW.


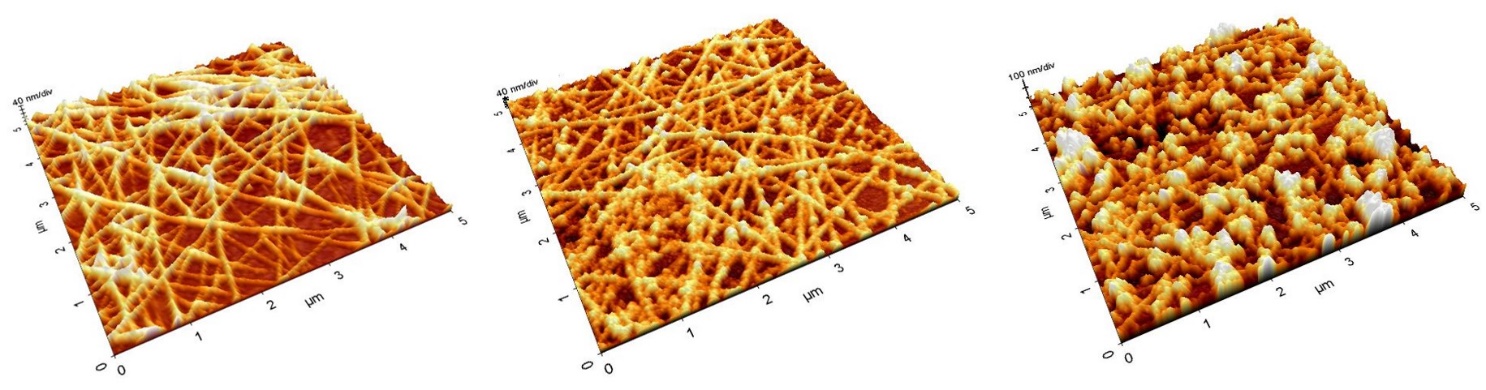


**Figure S6**. AFM image of pristine and EPD processed Ag NW. (a) the pristine Ag NW, EPD processed Ag NW at (b) 2.5V/cm and (c) 5V/cm.


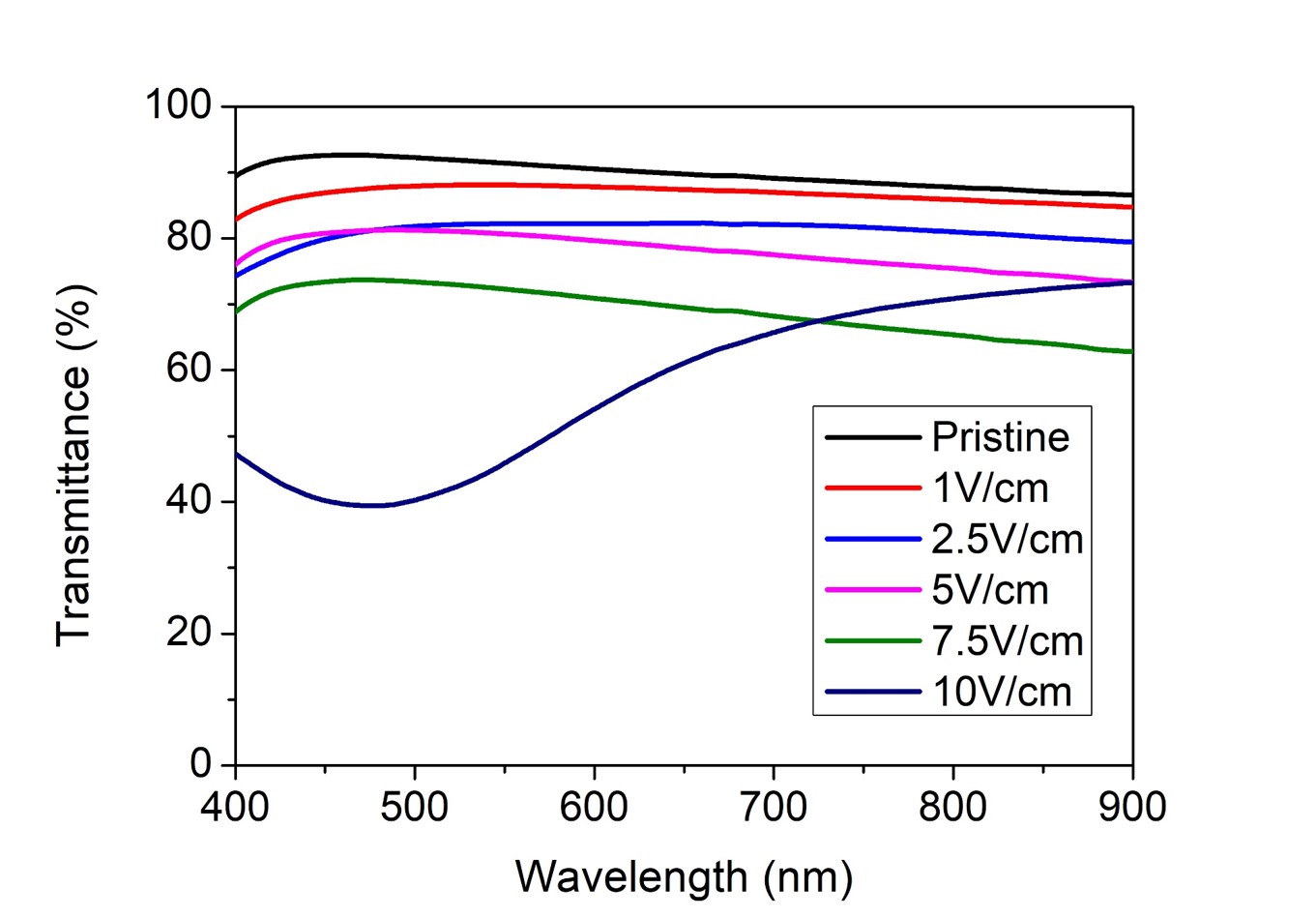


**Figure S7**. Transmittance spectra of the pristine and EPD-processed Ag NW.


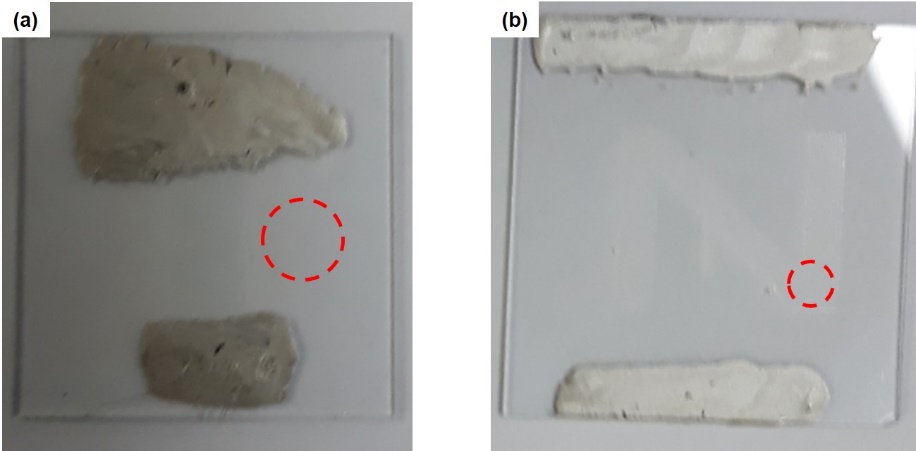


**Figure S8**. Optical photograph of patterned Ag NW. (a) the embossed line and (b) the engraved ‘N’ shape of patterned Ag NW. The red dashed areas present the ablated regions.


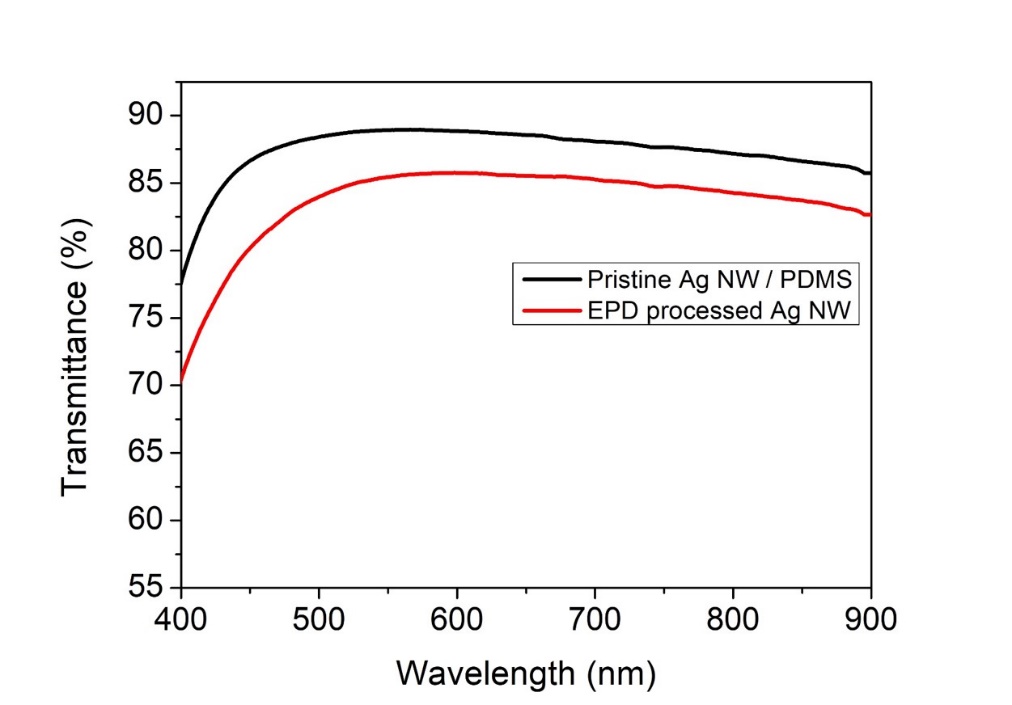


**Figure S9**. Transmittance spectra of the pristine Ag NW/PDMS and EPD-processed Ag NW at 1V/cm for 10min.


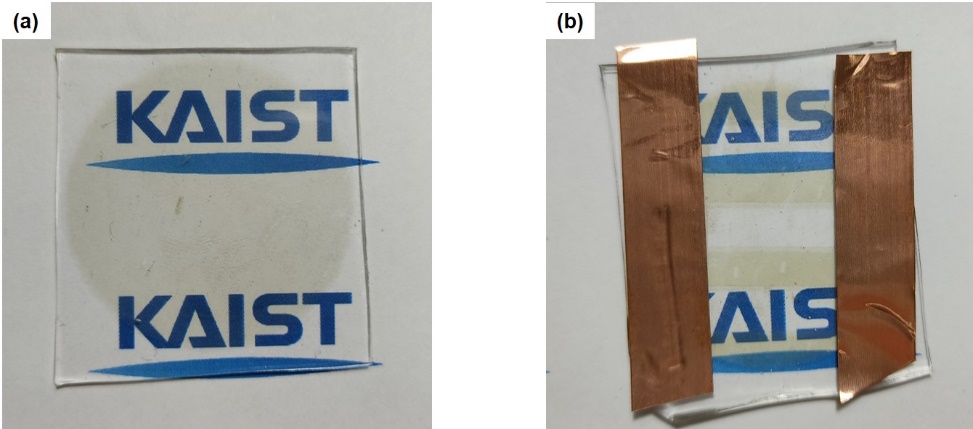


**Figure S10**. Optical photograph of patterned Ag NW/PDMS. (a) the transferred Ag NW on PDMS and (b) the patterned Ag NW/PDMS.


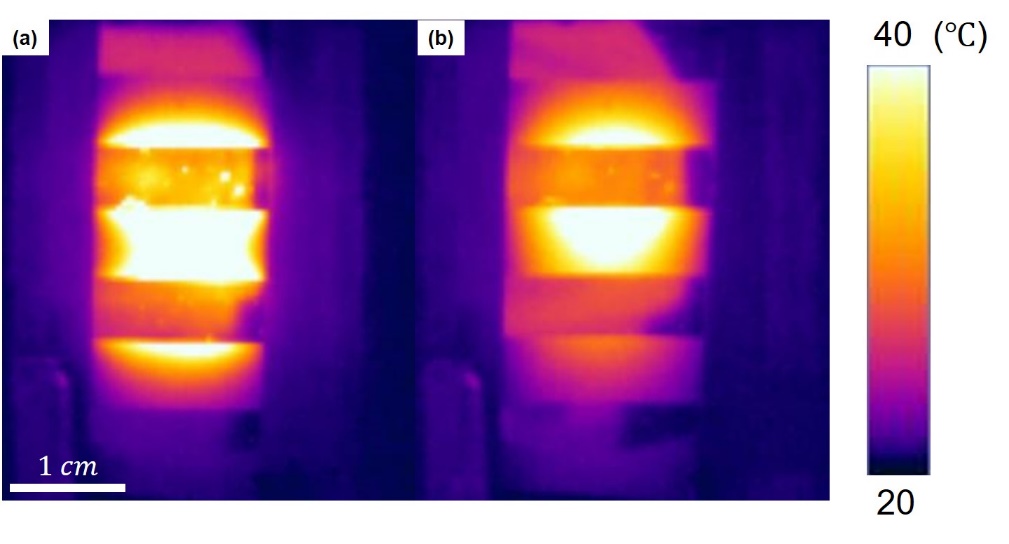


**Figure S11**. The infrared (IR) image of the patterned Ag NP decorated Ag NW/PDMS. Temperature distribution of patterned Ag NW with (a) 0% strain, (b) 20% strain condition.
